# Supplementary material for: Bayesian refinement of protein structures and ensembles against SAXS data using molecular dynamics
Source: PLoS Comput Biol. 2017 Oct 18;13(10):e1005800. doi: 10.1371/journal.pcbi.1005800 (PMC5662244; doi:10.1371/journal.pcbi.1005800)
Supplement: S1 Archive — (BZ2) [file pcbi.1005800.s011.tar.bz2 › gromacs_4.62_bayesian_saxs_refi/share/html/online/options.html]

Options


## Options

**VERSION 2.0**  

- Optional files are not used unless the option is set, in contrast to
  non optional files, where the default file name is used when the
  option is not set.

  All GROMACS programs will accept file options without a file extension
  or filename being specified. In such cases the default filenames will
  be used. With multiple input file types, such as generic structure
  format, the directory will be searched for files of each type with the
  supplied or default name. When no such file is found, or with output
  files the first file type will be used.

  All GROMACS programs with the exception of mdrun,
  trjcat and eneconv check if the command line options
  are valid. If this is not the case, the program will be halted.

  - All GROMACS programs have 4 hidden options:

    | option | type | default | description |
    | --- | --- | --- | --- |
    | **-[no]hidden** | bool | yes | [hidden] Print hidden options || **-[no]quiet** | bool | no | [hidden] Do not print help info || **-man** | enum | html | [hidden] Write manual and quit: no, html, tex, nroff, java, ascii or completion || **-[no]debug** | bool | no | [hidden] Write file with debug information |

    - Enumerated options (enum) should be used with one of the arguments
      listed in the option description, the argument may be abbreviated.
      The first match to the shortest argument in the list will be selected.

      - Vector options can be used with 1 or 3 parameters. When only one
        parameter is supplied the two others are also set to this value.

        - All GROMACS programs can read compressed or g-zipped files. There
          might be a problem with reading compressed .xtc,
          .trr and .trj files, but these will not compress
          very well anyway.

          - Most GROMACS programs can process a trajectory with less atoms than
            the run input or structure file, but only if the trajectory consists
            of the first n atoms of the run input or structure file.
